# Mycobacterium tuberculosis mixed infections and drug resistance in sub-Saharan Africa: a systematic review

**DOI:** 10.4314/ahs.v22i1.65

**Published:** 2022-03

**Authors:** Lisa Nkatha Micheni, Serawit Deyno, Joel Bazira

**Affiliations:** 1 Department of Microbiology, Mbarara University of Science and Technology, Box 1410 Mbarara, Uganda; 2 Department of Microbiology and Immunology, Kampala International University Western Campus, Box 71, Bushenyi, Uganda; 3 Department of Pharmacology, Mbarara University of Science and Technology, Box 1410 Mbarara, Uganda

**Keywords:** Tuberculosis, mixed infection, drug resistance

## Abstract

**Background:**

Sub-Saharan Africa, is a region that records high rates of TB infection. Mycobacterium tuberculosis mixed strain infection, especially when the strains involved are of different susceptibilities, is an area of great interest because it is linked with an increased risk of treatment failure and transmission of resistant strains within the population. This paper reviewed original studies that reported MTB mixed infection and heteroresistance in the region between 2010 and 2020 to understand the extent of mixed strain infection and heteroresistance in the region. This information is very critical in the control of TB and ending the TB epidemic by 2035 as per the World Health Organization's vision.

**Methods:**

pubmed, Scopus, JSTOR, AJOL, and Google Scholar databases were searched through both key terms and subject headings. The literature was screened, assessed for the quality and evidence synthesized.

**Results:**

Eighteen original articles were included in this review after having met the inclusion criteria. The frequency of mixed strain infection reported in these studies varied between 2.8% and 21.1% while drug resistance range between 0.06% to 19% depending on the study design and the drug susceptibility screening technique utilized. The majority of the studies (50%) utilized Spoligotyping in conjunction with MIRU-VNTR typing in the detection of mixed infections.

**Conclusion:**

Despite the scarcity of data on mixed infections and heteroresistance in sub-Saharan Africa, various studies have revealed that these conditions are frequent in the region than previously thought. Given the evidence of the effect of mixed infections on drug resistance and treatment outcome, we conclude that mixed infection is an unavoidable topic for future studies.

## Introduction

Tuberculosis (TB), was declared a global health epidemic in 1993 by the World Health Organization (WHO) and up to date it continues to be a major public health concern^1^. It is associated with substantial morbidity and mortality with approximately 1.45 million deaths and 10 million newly diagnosed cases annually,^2^,^3^ making it the leading cause of death due to a single infectious agent worldwide^3^. TB infections were historically thought to be as a result of a single strain and any recurrence was assumed to be due to reactivation of the same strain that caused the first episode^4^. Infection duto multiple strains in a patient at a single point in time was barely considered. However, in the mid-1970s it was discovered, using phage typing, that different strains of Mycobacterium tuberculosis (MTB) could infect a patient at a single point in time^5^.

MTB mixed strain infection is described as a disease state in which a patient harbours more than one MTB strain at the same time either as a result of a single transmission involving multiple distinct strains or as a result of multiple transmission events^6^. Based on the information available from strain genotype databases such as http://www.ncbi.nlm.nih.gov/BLAST/mycobacterium.html, there are tens of thousands of MTB strains that are in existence and capable of infecting humans^7^. These strains are largely categorized into eight distinct genetic lineages (designated as L1 to L8)^7^,^8^. The different lineages are not only associated with particular geographical areas, but they also have distinct pathogenic characteristics that influence disease transmission, treatment outcomes, and antimicrobial drug resistance^9^–^11^. For instance, Lineage 1 (Indo-Oceanic) strains are known to be moderately virulent and are common in the Philippines, East Africa (also reported in Djibouti), and the Indian Ocean's rim while Lineage 2 (east Asian) is extremely virulent, more drug-resistant than other lineages and is predomint in Russia, East Asia and Central Asia^12^,^13^. Lineage 3 strains are concentrated in central Asia and East Africa while lineage 4 (Euro-American) are found in Europe, America, Africa, and the Middle East. Lineage 7 was recently discovered in Ethiopia where it is predominant^14^ while lineages 5,6, are highly restricted in West Africa^15^. Lineage 8 (L8) consists of strains specifically adapted to infect domestic and wild animals but are capable of causing disease in humans^16^–^18^. These L8 strains include, Mycobacterium bovis (cattle) which currently accounts for 3 to 16% of all TB cases in sub-Saharan Africa^19^–^21^ and Mycobacterium caprae (sheep and goats) causes approximately 2.8% of all TB cases in Africa^17^,^22^.

The advent of molecular genotyping techniques such as Mycobacterial Interspersed Repetitive Unit-Variable Number Tandem Repeat (MIRU-VNTR)^23^, Spoligotyping^24^ and Whole Genome Sequencing (WGS)^25^, has enabled the differentiation of these strains and detection of mixed infection in a patient at a single point in time. Mixed strain infection is more common in patients who live in areas with high infection pressure, regardless of whether they are immunocompetent or immunosuppressed^26^–^30^. Infection pressure hereby described as the number of MTB microbes and their ability to infect people in the community, meaning that when infection pressure is high, there are many microbes and people are much more likely become infected^28^. Studies have shown that approximately, 10%–20% of individuals living in areas with a high TB burden are infected with more than one strain of MTB^6^,^31^. A metagenomics study conducted by Kay et al., (2015) revealed a high rate of mixed infection in Europe during the 18^th^ century when the incidence of TB was high30. Another study conducted in South Africa, a country with a heavy TB burden, found high proportions of TB mixed infection of up to 54 %^32^. Despite a scarcity of data on mixed infection from countries with moderate TB burden, high proportions of mixed infection have also been identified^33^–^35^. For example, a study conducted in Iran, a country with a moderate rate of TB, revealed that 27% of Iranian TB patients had mixed infection^13^.

Mixed strain infection has have been associated with poor treatment outcomes and treatment failure^28^,^36^–^39^ especially if the infection is due to strains with different susceptibility patterns (sensitive and resistant strain). This condition herein referred to as heteroresistant, has been shown to play a key role in changing the drug susceptibility patterns^15^, ^20^–^23^. However, detection of heteroresistance can be difficult using the routine laboratory tests. For example, the GeneXpert Cepheid test, has been shown to have a low sensitivity in detecting mixed infection and a high false-negativity rate in detecting rifampin (RIF) resistance in cases of mixed infection^44^. This test can only reliably detect RIF resistance in clinical specimens when the MTB resistant subpopulation is > 50% of the bacilli in a sample (45–47) at a Ct value of 23.62 48. Other assays such as Line probe assay and high-resolution melting analysis can detect more than 50% of heteroresistance^26^–^28^. Delayed diagnosis of mixed infection and/or heteroresistance can increase the risk of treatment failure and encourage transmission resistant strains, mainly in high-TB-burden areas^52^ thus impeding TB control. Understanding the role of mixed infection on drug resistance and the magnitude of mixed infections in our community is essential if we are to end the TB epidemic by 2035 as envisioned by the World Health Organization's^2^. This review examines the available data on MTB mixed infection and heteroresistance in sub-Saharan Africa, and examines the association of mixed infection and drug resistance.

## Methods

### Search Strategy and Selection Criteria

The Preferred Reporting Items for Systematic Reviews and Meta-Analyses (PRISMA) guidelines^52^ were followed. A systematic advanced search of original articles published in English focusing solely on data dating from 2010 to 2020 in various online databases such as Medline (PubMed), JSTOR, AJOL and Google Scholar (additional search) was carried out. Search key terms included ‘tuberculosis’ ‘mixed infections’ and ‘drug resistance’ as described in Table 1. To ensure that we did not overlook any original papers that addressed the topic of mixed infection and heteroresistance, we widened our search terms to include terms such as “multiple strains,” “heterogeneous,” and “polyclonal,” “multidrug-resistant” and “antimycobacterial drug resistance” which are often used as alternate terms were also used. Titles and abstracts were screened for relevance, and subsequently reviewed the full texts of potentially pertinent manuscripts. Studies were eligible for inclusion if they were carried out on humans, used original datand if they reported evidence of mixed infection and/or drug resistance in any region within sub-Saharan Africa. Drug resistance studies that did not provide evidence of heteroresistance and information on the genotyping tool used for drug resistance analysis were excluded from the analysis. Those studies carried out in multiple countries were only included if the results were stratified by country.

**Table 1:** The database searches

Databases	Search terms	Results
PubMed	Filters: Humans; 2010–2020 #1 [MeSH Terms] tuberculosis OR "Mycobacterium tuberculosis" OR Tb #2 [All Fields] "mixed infection" OR "multiple strains” OR heterogenous OR polyclonal #3 [All Fields] "drug resistant" OR "multidrug resistant" **#1 AND #2 AND #3**	2,561
JSTOR	Limits: Academic content: Journals; Language: English; Date range 2010 to 2020; #1 (tuberculosis OR Tb OR “Mycobacterium tuberculosis”) #2 (“mixed infection” OR "multiple strains" OR heterogenous OR polyclonal) #3 (""drug resistant" OR "multidrug resistant") **#1 AND #2 AND #3.**	1,044
AJOL	#1 (tuberculosis OR tb OR "Mycobacterium tuberculosis") #2 ("mixed infection" OR "multiple strains" OR heterogenous OR polyclonal) #3 ("drug resistant" OR "multidrug resistant") **#1 AND #2 AND #3.**	173
Google Scholar	Limits: Item type: Article; Articles with all words; Date range 2010 to 2020 #1 tuberculosis AND mixed infection AND drug resistance	185*

### Data extraction

Data from selected studies were extracted using an Excel spreadsheet developed for this purpose. The extracted data include; the identity of the study (title, first author's last name and publication year), country of the study, number of samples collected and analyzed, a laboratory technique used for analysis, rate of mixed infection and TB drug-resistant cases reported. Thereafter, three categories were developed for the analysis of our findings: mixed strain infection, TB drug resistance and an association of mixed strain infection with TB treatment/drug resistance.

## Results

**Fig 1:**
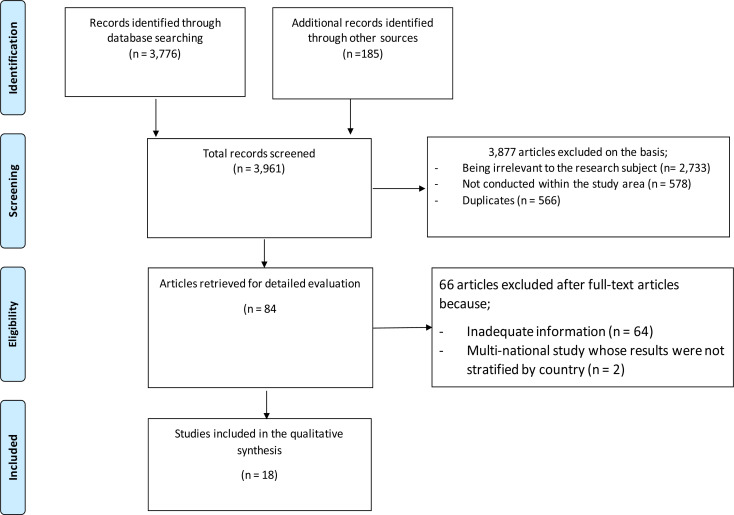
PRISMA study selection flow diagram for screened, excluded and included studies

The initial search identified 3,961 articles, which were reduced to 3,395 after eliminating duplicates. After 88 reviewing titles and abstracts an extra 3,311 were excluded as irrelevant for the research topic. On full-text examinations, 18 articles were selected as meeting inclusion criteria (figure 1), in which two sets of papers had similar results, were conducted in the same area, and were conducted in the same timeframe by similar researchers, and thus only one paper from each set was considered. Muwonge et al., 2013 53,54 conducted the first set of studies in Uganda, while the second set was conducted in Botswana^29^,^44^. Of the original eighteen articles, fourteen of the studies reported cases of heteroresistance, four articles reported cases of mixed infection while the other six articles reported cases of polyclonal resistance or mixed infection alone. Of the four studies that showed evidence of drugesistance on different MTB genotypes, one was a multinational study^55^. Based on the results of the 18 articles, the rate of mixed strain infection ranged between 2.8% to 51.0% while MTB drug resistance was between 0.06% (for ethambutol resistant) and 48.9% (MDR). (Table 2).

**Table 2:** Studies conducted within Sub-Saharan Africa on mixed tuberculosis infection and/or drug resistance; January 2010–December 2020

Sno.	Study	Country	No. of initial patients recruited/ samples	No. of samples analyzed	No. of cases with Mixed infection	%age rate of Mixed infection	No. of DR cases	% age rate of DR	Technique used
1	Mulenga et al., (2010) (60)	Zambia	361	152	5	3.2%	-	-	Spoligotyping, 15-locus MIRU-VNTR
2	Dickman et al., (2010) (61)	Uganda	113	113	11	7.1%	-	-	15-locus MIRU-VNTR
3	Mallard et al., (2010) (62)	Malawi	72 patients/ 160 sputum samples	256	2	2.8%	-	-	IS6110-RFLP, Spoligotyping
4	Hanekom et al., (2013) (63)	South Africa	535	206	31	15%	-	-	PCR methods
5*	Shin et al., (2015) (29)^*1^	Botswana	370	370	37	10%	55 patients	14.9% MDR	24-locus MIRU-VNTR
	Zetola et al., (2014) (44)^*1^	Botswana	370	370	37	10%	55 patients	14.9% MDR	Spoligotyping, 24-locus MIRU-VNTR
6*	Muwonge et al., 2013 (57)^*2^	Uganda	344	74	8	11.1%	(13DR; 2 MDR)	19% DR; 3% MDR	Spoligotyping, 15 loci MIRU-VNTR
	Muwonge et al., (2013) (58)^*2^	Uganda	344	74	12	11.1%	(13DR; 2 MDR)	19% DR; 3% MDR	Spoligotyping, 15-locus MIRU-VNTR
7	Zetola et al., (2014) (36)	Botswana	539	475	33/475	7%	483/539	88.1% MDR	DST phenotypic methods
8	Cohen et al., (2011) (64)	South Africa	240	56	5	9%	12	21% MDR	24-locus MIRU-VNTR
9	Ssengooba et al., (2015) (65)	Uganda	66	51	2	4%	3; 0	0.06% ethambutol	Spoligotyping, resistant 24-locus MIRU-VNTR
10	Guerra-Assuncąõ et al., (2015) (66)	Malawi	1933	1471	2	0.01%	-	3.7% isoniazid resistant	IS6110-RFLP, WGS
11	Cohen et al., (2016) (67)	South Africa	500	436	92	21.1%	-	3.79% MDR	MIRU-VNTR
12	Shin et al., (2018) (68)	Botswana	299	260	25	9.6%	30	11.5% MDR	24-locus MIRU-VNTR
13	Anselmo et al., (2019) (69)	Mozambique	79	79	4 distinct spoligotypes	-	14	17.72% MDR	Spoligotyping, Line probe assay
14	Bazira et al., (2011) (70)	Uganda	167	125	79 distinct spoligotypes	-	8	6.4% MDR	Spoligotyping, RD analysis
15	Kidenya et al., (2019) (71)	Tanzania	78	74	6 distinct spoligotypes	-	6DR 1 MDR	8.1% DR; 1.4% MDR	Spoligotyping WGS
16	Solo et al., (2020)(72)	Zambia	274	274	Various genotypes	-	134 MDR;	48.9% MDR	Spoligotyping, LSP, WGS
17	Kigozi et al., (2018) (73)	Uganda	97	97	Various genotypes	-	38 MDR	39.2% MDR	WGS
18	Kateete et al., (2019) (59)	eSwatini, Somalia and Uganda	80	80	Various genotypes	-	40 MDR; 24 XDR	50% MDR; 30% XDR	WGS

## Discussion

### Mycobacterium tuberculosis mixed infection

Despite the scarcity of data on MTB mixed infections in Sub-Saharan Africa we identified some studies from nine countries that reported mixed infections among patients. Table 2 shows these studies and the prevalence rates of mixed strain infections reported which ranges between 2.8% (58) and 21.1% 63. Detection of mixed strain infection in TB patients is greatly influenced by the molecular technique utilised to differentiate between the strains. Different molecular techniques ranging from IS6110 restriction fragment length polymorphism (RFLP) typing to Whole Genome Sequencing (WGS), are in use for the detection of mixed strain infection. In the last two decades, the IS6110 RFLP typing technique, which is based on analysis of the variation in the copy number of IS6110 unique insertion sequence of MTB 70, has significantly been applied in the detection of mixed strain infections and is considered the gold standard 71,72. This technique is highly discriminatory and produces consistent and repeatable results. However, it is time-consuming since it requires large amounts of DNA that can only be obtained through extensive cultures and suffers from problems of interpretability and portability of the complex banding patterns^73^. Furthermore, it does not provide adequate discrimination among isolates with low (≤ 5) IS6110 copy numbers, a problem that is only partially addressed by using Spoligotyping as a secondary method^74^,^75^. Spoligotyping is a cheap and robust method^76^, but it has a low discriminatory power hence generally not recommended for use on its own in the study of mixed infections^77^. Analysis of the Mycobacterial Interspersed Repetitive Units (MIRUs) that are located mainly in intergenic regions spread throughout the MTB genome is another technique that is in use. This technique has been shown to have high discriminatory power, quick turnaround time and reliable identification of mixed infections^54^,^71^,^78^,^79^. The use of this technique as a first-line tool in combination with Spoligotyping has been shown to provide sufficient discrimination for large-scale prospective MTB genotyping in the majority of cases^73^. The original MIRU-VNTR typing systems made use of limited sets of loci^4^, ^6^–^9^ hence low discriminatory power^80^. However, with time there was a development of more reliable systems that analyses extensive sets of 11 to 24 VNTR loci^23^,^75^,^81^–^83^. A system based on analysis of the 12 MIRU-VNTR loci is currently the most commonly epidemiological tool in use technique^78^,^81^,^84^, however, alternative sets of MIRU-VNTR loci such as 15 and 24 loci that enhance the discrimination of unrelated isolates are also in use^23^,^72^,^85^,^86^. Whole Genome Sequencing (WGS) analysis, is another molecular technique that was invented two decades ago. This technique provides high-resolution data on mixed infection as it gives a detailed overview of the different strains of mixed infection that cannot be evaluated using other methods^25^,^87^. Its major drawbacks that can restrict its use especially in many developing nations is its high operational cost and difficulties in interpreting the generated data88,89. Studies presented in this review utilized more than one technique to identify mixed infection whereby half of them (10/20;50%) utilized Spoligotyping as one of the typing methods. Five of these studies utilized Spoligo typing in conjunction with MIRU-VNTR and a rate of 4% (61) to 11.1%^53^ of mixed infection was identified. One study that utilized IS6110-RFLP in conjunction with WGS detected a 0.01% rate of mixed infection^62^ while a study that utilized MIRU-VNTR alone identified a 21.1% rate of mixed infection^63^.

Detection of mixed strain infection is also influenced by the sampling approach. Prior studies have shown that detection of mixed infections vary depending on whether one is either using direct sputum samples or cultures^90^. According to Farman farmaei and his colleagues, genotyping done on cultures could result in bias since the use of several culture collections or culture on numerous media can reduce the likelihood of identifying mixed infections. It has also been shown that the type of culture media used has a significant impact on the detection of mixed infections, whereby, LJ media was more sensitive than other media such as MGIT media^59^,^90^ and that the clonal composition changes significantly after culture^91^. Furthermore, evidence of different strains in a clinical specimen is highly dependent on the collection and handling/processing of the sample. For instance, collecting a single, low-volume sputum sample containing a large amount of upper airway secretions may substantially reduce the chance of detecting a mixed infection^33^. Shamputa et al., showed that increasing the number of sputum samples collected increases the likelihood of detecting mixed infections and that analyzing several sputum samples from various cavities within the lung could profoundly increase the likelihood of detecting a mixed infection. Studies reviewed here, relied on the collection of sputum samples from multiple pre-treatment specimens^61^ and typing of cultures from smear-positive individuals, collected serially over time^33^,^44^,^63^,^64^ and thus increased the sensitivity of their assay.

### Mycobacterium tuberculosis drug Resistance

Drug resistance is a situation that is of great concern since it poses a big challenge in the control of TB and ending of the TB epidemic by 2035 as envisioned by the WHO (2). This review identified fourteen studies that showed the presence of either one or more types of drug resistance. These include monoresistance, multidrug-resistant TB (MDR-TB) or extensively drug-resistant TB (XDRTB). Monoresistance is defined as the resistance of MTB to one antimycobacterial drug such as rifampin (RIF) or isoniazid (INH) for example RIF- resistant TB (RR-TB) -describes MTB that is susceptible to INH but resistant to RIF. MDR-TB is defined as concurrent resistance of MTB to first-line drugs rifampicin (RIF) and isoniazid (INH). XDR-TB is defined as MDR-TB strains with additional resistance to a fluoroquinolone and at least one of the injectable aminoglycosides^92^,^93^ and was first identified in 2006^94^. Table 2 shows the studies that reported drug resistance/heteroresistance and the prevalence rate. From these studies the reported rate of monoresistance ranged from 0.06% for ethambutol61 to 3.7% for isoniazid^62^ while the MDR cases ranged between 1.4% (67) to 48.9% 68. It is worth noting that the study by Kateete et al., (2019) that reported a 50% and 30% rate of MDR and XDR respectively, analyzed only samples that had been deemed as drug resistance^55^ hence may not necessarily reflect the actual prevalence at the community level. While much progress has been made in the recent past to understand the drug resistance situation in sub-Saharan Africa^47^,^95^–^98^, adequate data is still lacking due to weak surveillance and diagnostic capabilities^40^. For instance, rifampicin and isoniazid resistance is well monitored and described in most countries, either through continuous surveillance or periodic surveys unlike resistance to ethambutol and Pyrazinamide (PZA), the other two first-line drugs, which are not routinely monitored and thus poorly described^99^.

### Association of mixed infection with TB treatment/drug resistance

Despite the limited data on the effect of mixed infections (particularly when strains with distinct drug susceptibility patterns are involved) and limited sensitivity of current methods for detecting mixed infections, researchers have been able to identify deleterious effects of mixed infections for individuals who are affected and potential consequences for disease control. Using strain-specific PCR to detect mixed infections and detailed treatement data, Van Rie and his colleagues demonstrated that in patients harbouring both MDR and susceptible strains, the MDR strain population was able to survive and grow during a treatment with first-line antibiotics and upon a switch to second-line regimens, the susceptible strains were able to reemerge^100^. Apart from demonstrating MDR strains' relative fitness deficits in these patients, the findings revealed that mixed infections with strains of different resistance phenotypes could compromise treatment outcomes when using standard treatment regimens. Other studies have also shown that drug susceptibility patterns change through the presence or absence of antibiotic pressure, which determines the dominant growth of the coinfecting strain^6^,^36^,^101^. This implies that mixed infections with resistant strains could have poor clinical outcomes because the existence of any underlying drug resistance greatly decreases the efficacy of standardized antibiotic regimens and increases the risk of acquired resistance. Furthermore, there is a possibility of a reemergence of the drug-resistant strains when another regimen is introduced as a result antibiotic pressure^6^,^28^ or increase the risk of the drug-resistant strain acquiring additional drug resistance mutations^28^ In addition to the ability of multiple-strain infections to impair treatment efficacy, it has been hypothised that superinfection can occur some time after the primary infection, resulting in disease progression and endogenous reactivation of the primary infection. This means that the primary infection cannot provide immunity against a secondary infection^38^.

Mixed infections not only have an impact on the patient, but also on the community by negatively impacting on antituberculosis interventions and promoting transmission of resistant strains. Colijn et al.^102^, for instance, used a simple mathematical model to demonstrate how TB mixed infections promote the spread of resistant strains when interventions which affects drug-resistant and drug-sensitive strains differently such as isoniazid preventive therapy (IPT), are used, result in an increased ability of the resistant strain to take over. Another model study by Cohen and his colleques^103^ on the possible effects of new tuberculosis vaccines, they discovered that in mixed infections, by allowing multiple strain types to coexist, there was possibility of eroding the expected benefits of the new vaccine that does not sufficiently cover all circulating strain types, since the vaccine woulds be more likely lead to strain replacement. Therefore, despite the much difficulty in detecting MTB strains with different susceptibility profiles especially using the routine culture-based drug-susceptibility techniques^28^ it is very crucial to determine MTB mixed infection in patients and detect heteroresistance to ensure an appropriate antibiotic therapy regimen is chosen and eventually improving the treatment outcome and reduce the opportunity community transmission of drug resistant strains.

## Conclusion

Despite the scarcity of data on mixed infections and heteroresistance in Sub-Saharan Africa, studies have revealed that these conditions are more frequent in the region than previously thought. Given the evidence of the effect of mixed infections on drug resistance and treatment outcome, we conclude that mixed infection is an unavoidable topic for future studies.

## Limitations

Our findings are limited by the relatively few number of studies conducted in sub- Saharan Africa and due to the large heterogeneity amongst the included studies, this prohibited us from conducting a meta-analysis.

## Abbreviations

DNA: Deoxyribonucleic acid
DR-TB: Drug resistant - tuberculosis
DST: Drug susceptibility tests
INH: Isoniazid
IS6110-RFLP: Insertion sequence 6110- restriction fragment length polymorphism
MDR-TB: Multidrug resistant - tuberculosis
MIRU- VNTR: Mycobacterial Interspersed Repetitive Unit - Variable Number Tandem Repeat
MTB: Mycobacterium tuberculosis
MTBC: Mycobacterium tuberculosis complex
PCR: Polymerase chain reaction
PRISMA: Preferred Reporting Items for Systematic Reviews and Meta-Analyses
PZA: Pyrazinamide
RIF: Rifampicin
RR-TB: Rifampicin resistant tuberculosis
TB: Tuberculosis
WHO: World health organization
WGS: Whole-genome sequencing
XDR-TB: Extensively drug-resistant – tuberculosis
XXDR-TB: Totally drug-resistant – tuberculosis
